# Field evaluation of *Nepeta cataria*–based biopreparation delivered via an automated spraying system for sustainable control of cattle ectoparasites under pasture conditions

**DOI:** 10.14202/vetworld.2026.2010-2022

**Published:** 2026-05-16

**Authors:** Gulzhan Yeszhanova, Rashit Uskenov, Saule Bostanova, Igor Tretyakov, Alibek Mutushev, Arman Mirmanov, Fariza Zhagipar, Lyudmila Lider

**Affiliations:** 1 Institute of Animal Science and Veterinary, Saken Seifullin Kazakh Agrotechnical University, Astana, Kazakhstan; 2 LLP Kazakhstan Technology Commercialization Center, Astana, Kazakhstan; 3 LLP Economic Policy Analytical Center, Astana, Kazakhstan; 4 LLP Scientific and Production Technological Center “Zhalyn”, Almaty, Kazakhstan; 5 Institute of Energy, Saken Seifullin Kazakh Agrotechnical University, Astana, Kazakhstan; 6 Organic Agrotechnology Engineering Center, Agrotechnical park Seifullin University, Saken Seifullin Kazakh Agrotechnical University, Astana, Kazakhstan

**Keywords:** automated spraying system, cattle, ectoparasites, digital livestock management, hematological safety, insecticidal biopreparation, *Nepeta cataria*, plant-based insecticide, sustainable parasite control

## Abstract

**Background and Aim:**

Ectoparasites are a major constraint to cattle productivity, welfare, and health under pasture conditions. Conventional chemical control methods are increasingly limited by the development of resistance, environmental contamination, and residue accumulation in animal products. Plant-derived insecticidal formulations combined with precision livestock technologies represent a promising alternative. This study aimed to evaluate the field effectiveness and safety of a *Nepeta cataria* L.–based biopreparation delivered through an automated spraying system for sustainable control of cattle ectoparasites.

**Materials and Methods:**

A total of 145 clinically healthy cattle maintained under uniform pasture conditions were included in a prospective field trial. The biopreparation contained *N. cataria* essential oil (0.20%) incorporated into a plant-based formulation and applied as a 5% aqueous solution using an automated radio-frequency identification-integrated spraying system. Animals received a cumulative dose of 500 ± 100 mL through repeated voluntary passages over 2–3 days. Laboratory bioassays evaluated concentration-dependent insecticidal activity against *Musca* spp., *Tabanidae*, and *Dermacentor* spp. Field efficacy was assessed using the abundance and occurrence indices on days 7, 14, and 21 post-treatment. Physiological, hematological, and biochemical parameters were monitored to assess safety.

**Results:**

Laboratory assays demonstrated significant concentration-dependent insecticidal activity, with mortality reaching approximately 86.0%–87.7% across the tested ectoparasites at higher concentrations, comparable to that of a synthetic reference insecticide. Under field conditions, complete suppression of detectable ectoparasite infestation was observed at days 7 and 14 following completion of the cumulative dose. Partial re-infestation occurred by day 21, with efficacy ranging from 66.7% to 88.6% depending on parasite species. Seasonal dynamics indicated high baseline infestation levels, particularly for *Musca* spp. and *Simulium* spp. All physiological, hematological, and biochemical parameters remained within reference ranges, confirming treatment safety despite minor statistically significant variations in selected indicators.

**Conclusion:**

Automated cumulative application of an *N. cataria*–based biopreparation provides effective short-term control of cattle ectoparasites under pasture conditions without adverse health effects. The integration of plant-derived insecticidal formulations with automated delivery systems represents a technologically feasible and environmentally sustainable strategy for ectoparasite management in grazing livestock. Further controlled studies are required to optimize dosing strategies and evaluate long-term efficacy and ecological impact.

## INTRODUCTION

Organic farming in the Republic of Kazakhstan has been actively developing since the early 2000s and is regulated by the Law “On Organic Production” (2015). Its objectives include improving food quality, conserving natural resources, and promoting environmentally sustainable livestock production. Within this framework, effective prevention of parasitic diseases, particularly ectoparasite infestations under pasture conditions, remains essential for maintaining animal health and productivity [1–3].

Ectoparasites represent a major veterinary and economic challenge in cattle production systems worldwide. More than 100 insect species interact with livestock in grazing environments, causing irritation, reduced productivity, impaired physiological condition, and economic losses [4]. Despite the high level of development of veterinary medicine, the problem of arachnoentomoses persists even in economically developed countries, including the United States, European Union countries, and East Asian countries, creating year-round invasive pressure [5]. Infestation can reduce live weight gain in young cattle by up to 40% and decrease milk fat content by 25–50% [6]. In addition to direct damage, blood-feeding insects act as vectors of infectious diseases and contribute to the spread of parasitic infections [7–10]. In several regions, including Northern Kazakhstan, horsefly-associated diseases, such as hypodermosis, are widespread, with high prevalence and significant epizootiological risk [11].

Control strategies primarily rely on synthetic insecticidal and acaricidal compounds; however, prolonged use of pyrethroids and macrocyclic lactones has resulted in increasing parasite resistance, environmental contamination, and residue accumulation in animal products [12–14]. These limitations are particularly critical in organic livestock production systems, where the use of chemical treatments is restricted. Consequently, plant-based insecticides and essential oils have gained attention as potential alternatives. Essential oils possess lipophilic properties, penetrate insect cuticles, and affect arthropod nervous systems, including inhibition of acetylcholinesterase [15, 16]. Among these, *Nepeta cataria* L. has demonstrated insecticidal and repellent activity against several ectoparasites in laboratory conditions due to its terpene and phenolic constituents [17–22]. However, the practical application of *N. cataria*–based formulations under pasture conditions remains insufficiently investigated.

In parallel, digital technologies and smart livestock systems are increasingly applied to automate animal management and improve monitoring of production processes [23–26]. Automated spraying systems enable stress-free treatment of animals and allow controlled delivery of veterinary preparations under field conditions. Despite these advancements, studies integrating plant-based insecticidal formulations with automated application systems for ectoparasite control in grazing cattle remain limited.

Although the insecticidal and repellent properties of *N. cataria* essential oil have been extensively demonstrated under laboratory conditions, there is a significant lack of field-based evidence evaluating its effectiveness under real pasture environments, where environmental variability, animal behavior, and parasite dynamics can substantially influence treatment outcomes. Furthermore, most previous studies have focused on single-dose or manually applied formulations, which do not reflect the practical conditions of livestock management. The integration of plant-derived biopreparations with precision livestock technologies, particularly automated delivery systems, remains largely unexplored. There is also a limited understanding of how cumulative dosing strategies, achieved through voluntary animal interaction with automated systems, influence treatment efficacy, persistence, and safety. In addition, data on the combined performance of plant-based formulations and digital livestock platforms under organic farming constraints are scarce, especially in regions such as Kazakhstan, where ectoparasite pressure is high and climatic conditions vary seasonally. These gaps highlight the need for comprehensive field evaluations that combine biological efficacy, technological feasibility, and animal safety.

Therefore, the aim of the present study was to evaluate the effectiveness and safety of controlling cattle ectoparasites using a biological preparation containing *N. cataria* essential oil delivered through an automated spraying system under pasture conditions. Specifically, the study sought to (i) assess the *in vitro* insecticidal activity of the formulation against major ectoparasite groups, (ii) determine field efficacy based on changes in Abundance Index (AI) and Occurrence Index (OI) following automated cumulative dosing, (iii) evaluate the operational performance of the automated spraying system in delivering controlled treatment under grazing conditions, and (iv) investigate the physiological, hematological, and biochemical responses of treated animals to confirm safety. The study also aimed to explore the feasibility of integrating plant-based insecticidal formulations with precision livestock technologies as a sustainable strategy for ectoparasite management.

## MATERIALS AND METHODS

### Ethical approval

All experimental procedures involving animals were conducted in accordance with high standards of biosafety and animal welfare. Experimental protocols complied with the Ethical Guidelines for the Use of Animals in Research (2019), approved by the National Committee for Ethics in Scientific Research in Science and Technology [27]. Animal keeping and use were approved by the Bioethics Committee of the Institute of Animal Science and Veterinary of the Saken Seifullin Kazakh Agrotechnical University (Astana, Kazakhstan; protocol No. 1, February 23, 2023). All procedures were performed under field conditions using low-stress handling and voluntary passage through the automated system to minimize animal discomfort. No animals were intentionally left untreated under peak ectoparasite pressure because withholding treatment was considered potentially detrimental to animal welfare.

### Study period and location

The study was conducted between May 2023 and October 2024 during two grazing seasons. Field investigations were carried out on the pastures of the North Kazakhstan Agricultural Experimental Station LLP (North Kazakhstan region, Akkaiyn district, Shagalaly village). Laboratory analyses were performed at the Institute of Animal Science and Veterinary of the Saken Seifullin Kazakh Agrotechnical University, Astana, Kazakhstan.

### Study design

A total of 145 clinically healthy cattle were included in the study. All animals were maintained under identical pasture conditions and individually identified using radio-frequency identification (RFID) ear tags integrated into the automated treatment system. The body weight of cattle included in the study ranged from 410 to 530 kg. Animals were adult individuals of comparable physiological status and were maintained under uniform pasture conditions. The study was designed as a single-group prospective field trial without a separate untreated control group. The study was conducted without an untreated parallel control group due to animal welfare considerations under high ectoparasite pressure during the grazing season. Leaving a subgroup untreated under peak infestation conditions was considered potentially detrimental to animal welfare. Laboratory bioassays were conducted prior to field application to determine effective concentrations. Post-treatment insect counts were performed on days 7, 14, and 21 under comparable environmental conditions. Each animal served as its own control for comparison of pre- and post-treatment infestation levels. The sample size (n = 145) corresponded to the total herd size and was considered sufficient to detect statistically significant differences at p < 0.05.

### *N. cataria* biopreparation

The biological insecticidal preparation used in the study was produced in an accredited laboratory in collaboration with the “Zhalyn” Scientific and Production Technology Center (Almaty, Kazakhstan; accreditation certificate KZAF8C69D99A9FF2C9, November 8, 2022). The formulation is protected by the U. S. patent “Biopreparation against ectoparasites of cattle and method of application thereof” [28]. The product was developed from a liquid-phase plant formulation obtained by pyrolyzing rice husk biomass and adding *N. cataria* essential oil. The plant base consisted of an aqueous emulsion of the oil fraction (boiling point 300–400°C) possessing antiseptic, bactericidal, and insecticidal properties. Rice husk biomass contained phenol (0.7%) and cresol (0.5%) of total volatile substances. *N. cataria* essential oil was obtained by hydrodistillation using a Clevenger apparatus (Himlaborpribor, Klin, Russia). The oil was added to the plant base at a concentration of 0.2% per 100 mL of formulation. For field application, the concentrated formulation was diluted with water to obtain a 5% aqueous working solution prior to automated spraying.

### Laboratory evaluation of insecticidal efficacy

The insecticidal activity of the *N. cataria*–based biological preparation was evaluated under controlled *in vitro* laboratory conditions against representatives of the main ectoparasite groups affecting cattle: flies (*Musca* spp.), horseflies (*Tabanidae*), and ticks (*Dermacentor* spp.). Working solutions were prepared at concentrations of 0.02%, 0.05%, 0.10%, 0.20%, and 0.40% (v/v). These concentrations were selected to determine the concentration–response relationship and to identify a range of effective levels for subsequent field application of the formulation. A synthetic reference insecticide, Cyflunite (cyfluthrin; Bayer AG, Leverkusen, Germany), was used as a positive control at a concentration of 0.2 mg/mL in accordance with the manufacturer’s instructions. A negative control group treated with distilled water was included to assess natural mortality.

Adult flies (*Musca* spp.) and horseflies (*Tabanidae*) were collected from pasture areas of the experimental farm and acclimatized under laboratory conditions for 24 h prior to testing. Ticks (*Dermacentor* spp.) were manually collected from naturally infested cattle. Prior to testing, insects were maintained at 22–25°C and 60–70% relative humidity. Each treatment concentration was tested in triplicate, with 20 individuals per replicate (total n = 60 per concentration per species). Filter paper disks (Whatman No. 1; Cytiva, Marlborough, MA, USA) were treated with 1 mL of working solution and allowed to dry at room temperature. Insects were placed in contact with treated surfaces inside glass containers under standardized laboratory conditions (22–25°C, 60–70% relative humidity). Control groups were treated with distilled water. The laboratory bioassay was performed using a standard contact-exposure method widely used in entomological studies, with minor adaptations for the tested species. Mortality was assessed after 24 h. Insecticidal activity was defined as the percentage mortality of exposed insects within 24 h after contact with treated surfaces. Mortality was determined by direct visual observation; insects were considered dead if no movement was observed after gentle mechanical stimulation. The percentage mortality was calculated as:

Mortality (%) = (Nd/Nt) × 100

where Nd is the number of dead insects and Nt is the total number of insects in each replicate. If mortality in the control group exceeded 5%, corrected mortality was calculated using Abbott’s formula. The mean corrected mortality was used as the indicator of insecticidal activity for statistical analysis.

### Automated insecticide application system

Insecticide application was performed using an automated skin-treatment system integrated with a stress-free animal-weighing platform (Patent of the Republic of Kazakhstan No. 8658) [29]. The system was installed in the watering area and enabled identification, weighing, and insecticide application during a single voluntary passage of the animal. The system consisted of mechanical, hydraulic, electrical, and software subsystems. Spray nozzles were mounted on a galvanized steel arch structure positioned above the weighing platform at a height of 1,730 mm above ground level and at distances of 30, 65, and 105 cm from the watering trough frame to ensure overlapping spray patterns and uniform body-surface coverage. The hydraulic subsystem included a working solution tank, pump (nominal capacity 18 L/min), distribution pipeline, solenoid valves (12 V), and stainless-steel fine-spray nozzles with an outlet diameter of 0.6 mm. Operating pressure was maintained at 5.0 ± 0.2 kg/cm². Each nozzle provided a discharge rate of 200–260 cm³/min with a spray angle of approximately 70°. Animal identification was performed using UHF RFID ear tags. The control algorithm verified treatment intervals and cumulative dose prior to valve activation. The delivered volume was calculated according to preset dosing parameters and the duration of the animal’s stay in the treatment zone. All spray events were automatically recorded in a centralized database.

### System calibration and verification

Prior to biological efficacy trials, the system was tested under field conditions to verify pressure stability, nozzle discharge rate, and dosing accuracy. The delivered volume per activation cycle was measured and compared with programmed values. RFID accuracy and valve response were also evaluated. The system demonstrated stable pressure maintenance and reproducible spray performance before initiation of efficacy assessments.

### Insecticidal treatment protocol

A 5% aqueous working solution was prepared by diluting 0.5 L of the concentrated formulation in 10 L of water. This dilution was selected based on laboratory concentration–response results demonstrating substantial insecticidal activity of the formulation, while also considering field-application feasibility, cumulative dosing requirements, and the avoidance of excessive exposure under pasture conditions. The solution was applied using the automated spraying system described above. During a single passage through the treatment zone (average duration 60 ± 10 s), approximately 150–200 mL of working solution was sprayed onto the animal’s body surface. The target cumulative dose was set at 500 ± 100 mL per animal. The automated system recorded each spray activation and accumulated the applied volume over repeated voluntary passages until the predetermined total dose was reached. The target cumulative dose (500 ± 100 mL per animal) was achieved within 2–3 days by repeatedly passing animals through the automated spraying system. The system recorded each activation and accumulated the applied volume until the programmed cumulative dose was reached. No additional treatments were administered after the target dose had been achieved. Day 0 was defined as the day when the programmed cumulative dose was completed for each animal. An alternative mode with increased consumption of working solution was tested; however, it did not improve insecticidal efficacy, confirming the adequacy of the selected dosage and application protocol under pasture conditions.

### Collection of insects

The collection of midges and zoophilic flies, as well as entomological observations, was carried out according to the method described by Gibb et al. [30]. The location and number of insects on the animals’ bodies were documented. Each observation session lasted 10 min per animal. An Olympus CX 23 microscope (Olympus, Tokyo, Japan) and an insect identification key were used to determine insect species. Zoo-parasitological indices were applied to assess the epizootic status of ectoparasite infestation in cattle [1, 23]. To ensure the reliability of AI measurements, insect counts were conducted under standardized field conditions. Each animal was individually restrained in a handling chute during counting to prevent movement and minimize insect transfer between animals. For winged and mobile insects, visual counts were performed systematically by examining predefined anatomical zones (head, neck, back, flanks, abdomen, and limbs) in a fixed sequence to avoid duplication. Observers were trained and the same personnel conducted repeated counts throughout the study to reduce inter-observer variability. To minimize double-counting or undercounting, only insects visibly attached to or actively feeding on the animal at the time of observation were recorded. Insects flying transiently around the animal without landing were not included. The short, standardized observation period (10 min) reduced the likelihood of repeated migration between animals. When group-grazing conditions made immediate counting difficult, animals were sequentially separated for individual assessment. OI was defined as the percentage of animals infested with ectoparasites relative to the total number of examined animals.

OI = (Np/n) × 100

where Np is the number of infested animals and n is the total number of animals examined. AI was calculated as the average number of parasites per animal:

AI = Par/n

where Par is the total number of parasites detected.

OI was expressed as a percentage, characterizing the proportion of infested animals in the population, whereas AI reflected the overall parasite burden per animal and was expressed as the mean number of parasites per animal, in accordance with generally accepted parasitological assessment methods. Treatment efficacy in the field trial was assessed based on changes in AI relative to day 0. Efficacy (%) was calculated as the percentage reduction in AI compared with the pre-treatment value using the following formula:

Efficacy (%) = [1 − (AIt/AI_0_] × 100

where AI_0_ is AI before treatment on day 0, and AIt is AI at days 7, 14, or 21.

For flying hematophagous insects, this endpoint reflects the reduction in the number of insects observed on animals during standardized counts and may represent a combined outcome of insecticidal and repellent effects under field conditions. Blood-feeding success (fed vs. unfed insects) was not specifically assessed; therefore, repellency was not calculated according to standard laboratory repellent evaluation protocols. The chosen field endpoint, AI reduction, is consistent with commonly applied parasitological assessment approaches in pasture-based studies.

### Monitoring schedule and environmental standardization

Entomological monitoring was conducted regularly throughout the grazing period from May to October. Seasonal observations were classified as spring (May–June), summer (July–August), and autumn (September–October). To minimize environmental variability, all counts were performed under comparable meteorological conditions: air temperature 20–28°C, wind speed below 5 m/s, and absence of precipitation. Observations were avoided during heavy rain, strong wind, or abrupt temperature fluctuations.

Counts were conducted during peak insect activity periods (08:00–11:00 and 17:00–20:00). When environmental conditions differed substantially, observations were postponed to ensure data comparability.

### Assessment of animal safety and physiological condition

Physiological parameters, including body temperature, respiratory rate, and pulse rate, and blood samples were collected before treatment and on day 7 after insecticidal application to assess short-term safety. Blood samples were collected from the jugular vein in the morning before feeding. Analyses were performed using Hemax 330 Vet (B&E Bio-technology Co., Ltd., Beijing, China) and BioChem FC 200 (High Technology, Inc., Walpole, MA, USA) automatic analyzers according to standard methods. Blood parameters were evaluated as indicators of systemic physiological response to treatment.

### Statistical analysis

Statistical analysis was performed using IBM SPSS Statistics version 26.0 (IBM Corp., Armonk, NY, USA). Normality of data distribution was assessed using the Shapiro–Wilk test. Differences between baseline and post-treatment values on days 7, 14, and 21 were evaluated using paired t-tests. For repeated comparisons across all time points, repeated-measures one-way analysis of variance (ANOVA) was applied. Results are presented as mean ± SEM. Differences were considered statistically significant at p < 0.05.

## RESULTS

Study of the effectiveness of the *N. cataria* biopreparation

The laboratory evaluation demonstrated a statistically significant concentration-dependent increase in mortality against all tested ectoparasite groups (Table 1).

**Table 1:** Laboratory insecticidal efficacy of the *N. cataria* biopreparation at different concentrations.

No.	Concentration (v/v, %)	Flies (*Musca* spp.), mean ± SEM	Horseflies (*Tabanidae*), mean ± SEM	Ticks (*Dermacentor* spp.), mean ± SEM
1	0.02	80 ± 4	70 ± 4	60 ± 3
2	0.05	80 ± 2	80 ± 4	70 ± 3
3	0.10	95 ± 3	85 ± 2	90 ± 2
4	0.20	100 ± 2	100 ± 2	100 ± 2
5	0.40	100 ± 2	100 ± 2	100 ± 2
–	Reference insecticide, Cyflunite, 0.2 mg/mL	95 ± 5	98.5 ± 4	85 ± 4

SEM = Standard error of the mean, n = 60 insects per concentration per species. Mortality was assessed 24 h after exposure.

In *Musca* spp., mortality increased from 78.0 ± 1.15% at 0.02% to 86.0 ± 1.15% at 0.40%. In *Tabanidae*, mortality rose from 69.0 ± 1.15% to 87.7 ± 1.20%, while in *Dermacentor* spp. it increased from 57.3 ± 1.45% to 87.7 ± 1.45%. The reference insecticide Cyflunite (0.2 mg/mL) (Bayer AG) demonstrated mortality rates of 89.0 ± 0.80% in flies, 91.0 ± 0.90% in horseflies, and 87.0 ± 1.10% in ticks. ANOVA confirmed statistically significant differences between treatment concentrations (flies: F = 4.2, p < 0.05, horseflies: F = 9.5, p < 0.01, ticks: F = 28.0, p < 0.001). Laboratory efficacy was assessed as 24-h mortality under controlled exposure conditions, whereas field efficacy was evaluated as suppression of infestation indices (AI and OI) on animals over time.

### Seasonal dynamics of cattle ectoparasites

As summarized in Table 2, the seasonal dynamics of cattle ectoparasites during the grazing period showed marked differences in both AI and OI among parasite groups.

**Table 2:** Species composition and seasonal distribution of cattle ectoparasites during the pasture period.

Parasite species	Spring (May) AI, mean ± SEM	Spring (May) OI (%)	Summer (July) AI, mean ± SEM	Summer (July) OI (%)	Autumn (September) AI, mean ± SEM	Autumn (September) OI (%)
Grazing flies (*Musca* spp.)	35 ± 0.5	100	66.4 ± 1.2	100	78 ± 1.5	100
Blood-sucking mosquitoes (*Culex* spp.)	8.5 ± 1.5	100	10.5 ± 1.3	100	13.6 ± 2.3	100
Midges (*Simulium* spp.)	43 ± 1.1	100	46 ± 0.5	100	39 ± 2.4	100
Horseflies (*Tabanus* spp.)	0	0	12.5 ± 0.5	33.3	15.5 ± 3.3	28
Lice (*Bovicola bovis*)	15.6 ± 1.5	50.5	11.4 ± 2.1	40.6	10.5 ± 2.5	53.6

AI = Abundance Index, OI = Occurrence Index, SEM = Standard error of the mean.

*Musca* spp. demonstrated the highest abundance throughout the grazing season. The AI increased from 35.0 ± 0.5 in spring to 66.4 ± 1.2 in summer and reached 78.0 ± 1.5 in autumn, while OI ranged from 92% to 98%. *Culex* spp. showed a seasonal increase in AI from 8.5 ± 1.5 in spring to 13.6 ± 2.3 in autumn, with OI rising from 70% to 88%. *Simulium* spp. remained abundant across seasons (43.0 in spring, 46.0 in summer, and 39.0 in autumn). *Tabanus* spp. were absent in spring but appeared in summer (12.5 ± 0.5) and increased in autumn (15.5 ± 3.3). *Bovicola bovis* exhibited relatively stable values, with AI ranging from 10.5 to 15.6 and OI from 37% to 51%. *Dermacentor* spp. demonstrated moderate seasonal variability, with the highest abundance observed in spring (AI = 12.0 ± 1.0, OI = 64%), followed by a decrease in summer (AI = 6.5 ± 0.8, OI = 42%) and a slight increase in autumn (AI = 9.8 ± 1.4, OI = 55%). This pattern reflects the characteristic seasonal activity of ixodid ticks during the grazing period.

ANOVA confirmed statistically significant seasonal differences in parasite abundance, particularly for *Musca* spp. (F = 18.4, p < 0.001), followed by *Culex* spp. (F = 6.2, p < 0.01) and *Simulium* spp. (F = 4.9, p < 0.05).

### Automated skin treatment system

**Figure 1:**
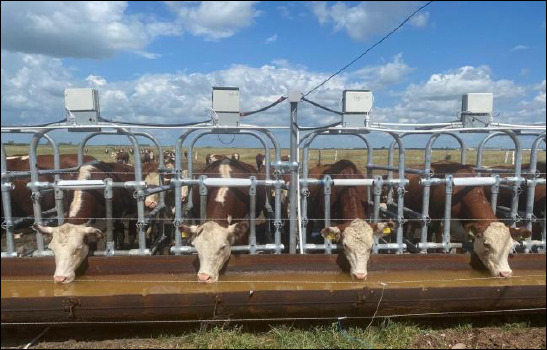
Automated insecticide application during voluntary passage of cattle through the watering area.

**Figure 2:**
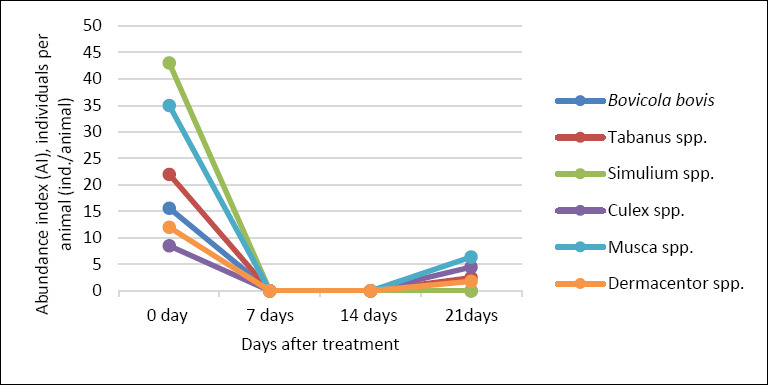
Dynamics of abundance index after treatment with an insecticidal biological product containing essential oil of *Nepeta cataria*.

**Figure 3:**
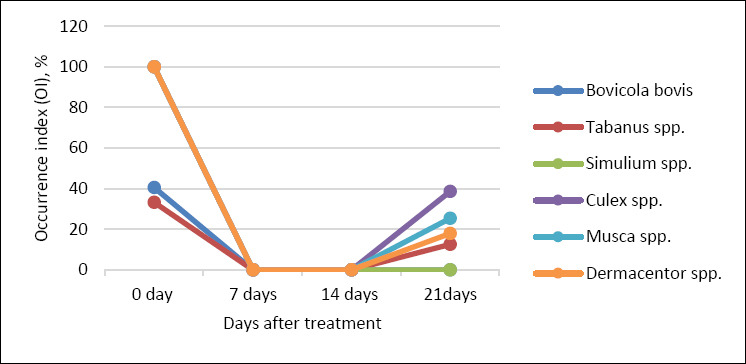
Dynamics of occurrence index after treatment with an insecticidal biological product containing essential oil of *Nepeta cataria*.

Insecticidal treatment was carried out using an automated skin treatment system integrated with a stress-free weighing platform (Patent of the Republic of Kazakhstan No. 8658). The system consisted of a passage structure equipped with controlled spray nozzles and an integrated weighing platform, enabling voluntary animal passage and automated monitoring of cumulative doses under pasture conditions. Field validation demonstrated that the herd was fully treated within 2–3 days. The target cumulative dose was 500 ± 100 mL per animal and was automatically reached during repeated voluntary passages through the treatment zone. No additional applications were performed after the programmed cumulative dose had been completed. Field efficacy of the *N. cataria* biopreparation. The process of spraying the working solution during the voluntary passage of animals through the watering area is presented in Figure 1. The duration of action of the biological product was assessed at 7-day intervals (Figures 2 and 3).

Figure 2 illustrates changes in AI following treatment with the *N. cataria*–based biopreparation. Before treatment (day 0), AI values varied among parasite species, with the highest levels recorded for *Simulium* spp. (43 ind./animal) and *Musca* spp. (35 ind./animal), followed by *Tabanus* spp., *Bovicola bovis*, *Dermacentor* spp., and *Culex* spp.

At 7 and 14 days after treatment, AI values for all parasite species decreased to 0, indicating the absence of detectable ectoparasite infestation during this period. By day 21, partial re-infestation was observed. AI values increased to 6.4 ind./animal for *Musca* spp., 4.5 for *Culex* spp., 2.5 for *Tabanus* spp., and 1.8 for *Dermacentor* spp., while *Simulium* spp. remained undetected.

These findings indicate a pronounced short-term field effect lasting approximately two weeks, followed by gradual reappearance of ectoparasites under pasture conditions.

Figure 3 presents changes in OI after treatment. Prior to application, infestation prevalence varied among species, reaching 100% for *Dermacentor* spp. and 40% for *Bovicola bovis*, while other species showed intermediate levels.

At 7- and 14-days post-treatment, OI decreased to 0% for all recorded ectoparasites, confirming absence of detectable infestation in the treated herd during this period. By day 21, OI values increased again, indicating renewed exposure and partial re-infestation under pasture conditions. The highest prevalence was recorded for *Culex* spp. and *Musca* spp., whereas *Simulium* spp. remained absent.

Treatment efficacy, expressed as percent reduction in AI relative to baseline, indicated complete suppression of detectable infestation at days 7 and 14 (Figure 2). At day 21, efficacy values remained high but decreased due to partial re-infestation, ranging from 66.7% to 88.6% depending on parasite species.

### Physiological, hematological, and biochemical indicators

The safety of the biological product was evaluated based on physiological, hematological, and biochemical parameters measured before treatment and 7 days after application. Physiological indicators are presented in Table 3.

**Table 3:** Physiological indicators of cattle before and after treatment.

Indicator	Temperature (°C), mean ± SEM	Heart rate (beats/min), mean ± SEM	Respiratory rate (breaths/min), mean ± SEM
Before treatment	37.92 ± 0.16	66 ± 0.63	18 ± 0.15
7 days after treatment	38.05 ± 0.06	60 ± 0.28	19 ± 0.05
Reference values	37.5–39.0	50–80	15–30

SEM = Standard error of the mean, n = 145 animals. Reference ranges correspond to physiological norms for adult cattle.

Body temperature did not differ significantly between observation points (ANOVA: F = 0.61, p = 0.44). Respiratory rate also showed no significant changes (F = 2.31, p = 0.15). Heart rate decreased from 66 ± 0.63 to 60 ± 0.28 beats per minute. The difference was statistically significant (ANOVA: F = 5.34, p = 0.04, t-test: p = 0.04). All physiological parameters remained within reference ranges. Hematological results are presented in Table 4.

**Table 4:** Hematological parameters before and after treatment.

Parameter	Reference range	Before treatment, mean ± SEM	7 days after treatment, mean ± SEM
Hemoglobin (g/L)	90–139	104.3 ± 0.91	107.8 ± 0.22
Erythrocytes (×10¹²/L)	5–10	6.47 ± 0.28	7.25 ± 0.026
Leukocytes (×10⁹/L)	5–16	11.92 ± 0.14	11.81 ± 0.03
Lymphocytes (%)	20–60	36.0 ± 0.23	39.6 ± 0.12
Monocytes (%)	4–12	4.3 ± 0.03	4.12 ± 0.06
Granulocytes (%)	30–65	60.0 ± 0.08	59.2 ± 0.027
Thrombocytes (×10⁹/L)	120–820	441.25 ± 0.39	459.6 ± 0.62

SEM = Standard error of the mean, n = 145 animals. Reference ranges correspond to physiological norms for adult cattle.

Hemoglobin concentration increased from 104.3 ± 0.91 g/L to 107.8 ± 0.22 g/L (t = 3.45, p = 0.018, ANOVA: F = 3.89, p = 0.04). Erythrocyte count increased from 6.47 ± 0.28 × 10¹²/L to 7.25 ± 0.026 × 10¹²/L (t = 2.78, p = 0.032, ANOVA: F = 4.12, p = 0.038). Lymphocytes increased from 36.0 ± 0.23% to 39.6 ± 0.12% (t = 4.02, p = 0.014, ANOVA: F = 5.21, p = 0.028). Platelet count increased from 441.25 ± 0.39 × 10^9^/L to 459.6 ± 0.62 × 10^9^/L (t = 3.12, p = 0.025, ANOVA: F = 3.66, p = 0.046). Leukocytes, monocytes, and granulocytes did not show statistically significant changes (p > 0.05). All values remained within reference ranges. Biochemical parameters are presented in Table 5.

**Table 5:** Biochemical blood parameters before and after treatment.

Parameter	Reference range	Before treatment, mean ± SEM	7 days after treatment, mean ± SEM
Total protein (g/L)	77–120	115.0 ± 0.43	118.3 ± 0.19
Albumin (g/L)	31.6–47.2	38.56 ± 0.03	37.8 ± 0.046
Glucose (mmol/L)	2.3–3.8	2.35 ± 0.013	2.4 ± 0.02
Blood urea nitrogen (mmol/L)	2.5–6.9	4.8 ± 0.09	4.65 ± 0.011
Calcium (mmol/L)	2.5–3.38	2.65 ± 0.12	2.8 ± 0.05
Phosphorus (mmol/L)	1.3–2.0	1.63 ± 0.37	1.58 ± 0.014
Cholesterol (mmol/L)	1.3–4.4	3.42 ± 0.19	2.93 ± 0.06
Creatinine (μmol/L)	55.8–160	102.3 ± 0.42	113.0 ± 0.36
Alkaline phosphatase (IU/L)	≤164	69.4 ± 0.63	71.1 ± 0.05

SEM = Standard error of the mean, n = 145 animals. Reference ranges correspond to physiological norms for adult cattle.

Total protein increased from 115.0 ± 0.43 to 118.3 ± 0.19 g/L (p = 0.028, ANOVA p = 0.041). Calcium increased from 2.65 ± 0.12 to 2.80 ± 0.05 mmol/L (p = 0.039). Cholesterol decreased from 3.42 ± 0.19 to 2.93 ± 0.06 mmol/L (p < 0.05). Triglycerides increased from 0.17 to 0.20 mmol/L (p = 0.022). Creatinine increased from 102.3 ± 0.42 to 113.0 ± 0.36 μmol/L (p = 0.014). Albumin, glucose, urea, phosphorus, and alkaline phosphatase did not change significantly (p > 0.05). All biochemical parameters remained within physiological reference ranges.

### Use of the automated spraying system

During the summer grazing period, animals were treated using the automated spraying system, and cumulative product consumption was monitored electronically. The system recorded spray activations and automatically accumulated the applied volume for each animal. Animals reached the programmed cumulative dose (500 ± 100 mL per animal) within several days through repeated voluntary passages. The average daily amount of product applied per animal varied due to differences in watering behavior and system activation frequency. Despite variability in daily exposure, efficacy assessment was conducted relative to the completion of the programmed cumulative dose (day 0), ensuring comparability of post-treatment evaluations across animals. Most animals received the intended cumulative dose within the programmed range.

## DISCUSSION

### Performance of the automated cumulative dosing system

The results demonstrate that the Smart automated spraying system enables controlled and individualized delivery of the insecticidal biological product, allowing animals to reach the programmed cumulative dose through voluntary passage within several days. This approach differs fundamentally from conventional single-application treatments, as it incorporates behavioral patterns of animals under pasture conditions and enables gradual accumulation of the active formulation without inducing handling stress. Although up to 150–200 mL of working solution could be delivered during a single passage through the spraying zone, the average daily consumption was lower due to variability in voluntary system activation [23–26]. Importantly, testing an alternative regimen with increased product consumption did not improve efficacy, indicating that the selected cumulative dose of approximately 500 mL per animal was sufficient under the field conditions studied.

Variability in system activation frequency was observed among animals due to differences in watering behavior. Nevertheless, the automated system was programmed to deliver a standardized cumulative dose of 500 ± 100 mL per animal, and efficacy assessment was conducted relative to completion of this cumulative exposure on day 0. Therefore, differences in daily activation frequency did not influence the final administered dose at the time of efficacy evaluation. However, environmental factors such as rainfall, solar radiation, and animal movement may influence the persistence of the formulation on the body surface, posing inherent challenges for pasture-based ectoparasite control strategies. Although the applied dose was not adjusted according to individual body weight, animals were within a relatively narrow weight range of 410–530 kg, which likely minimized variability in product distribution relative to body surface area.

### Differences between laboratory and field efficacy endpoints

The apparent discrepancy between laboratory mortality rates and field efficacy reflects differences in experimental endpoints. Laboratory bioassays measured direct-contact mortality under controlled exposure conditions, whereas field efficacy was assessed by suppression of ectoparasite infestation indices, including AI and OI. Under natural grazing conditions, the reduction in AI likely reflects a combination of direct insecticidal effects and behavioral avoidance of treated animals by ectoparasites. Consequently, direct quantitative comparison between laboratory mortality and field AI reduction is not methodologically equivalent. Digital smart livestock technologies are increasingly applied in modern animal husbandry to monitor animal health, productivity, and welfare [23–25]. However, their use for automated ectoparasite control remains insufficiently investigated. The present study demonstrates the feasibility of integrating automated cumulative dosing systems with plant-derived insecticidal formulations under practical pasture conditions.

### Biological activity of the *N. cataria*–based formulation

The biological activity observed for the *N. cataria*–based formulation is consistent with previously reported insecticidal and repellent properties of essential oils from this plant [19–22]. Essential oil activity has been shown to depend on concentration, formulation stability, and environmental exposure. In the present study, suppression of detectable infestation persisted for approximately two weeks, which is comparable with the expected persistence of volatile plant-derived compounds under field conditions. Importantly, the tested formulation consisted not only of *N. cataria* essential oil but also of a liquid-phase plant base derived from rice husk pyrolysis products. Pyrolysis-derived extracts are known to contain phenolic compounds, organic acids, and carbonyl derivatives that may exhibit intrinsic insecticidal or repellent properties. Therefore, the observed field efficacy cannot be attributed exclusively to the essential oil component. It is likely that the formulation’s biological activity reflects additive or potentially synergistic interactions between pyrolysis-derived compounds and essential-oil constituents. Phenolic derivatives may disrupt insect cellular membranes or interfere with neurosensory signaling, while interactions between formulation components may improve persistence, adhesion to the hair coat, or controlled release of volatile compounds. However, detailed mechanistic studies are required to clarify the specific contribution of each component.

### Practical relevance for sustainable ectoparasite management

Compared with conventional synthetic insecticides, plant-based formulations may offer several potential advantages. Although pyrethroids and macrocyclic lactones often provide high initial efficacy, their repeated use has been associated with the development of resistance in ectoparasite populations [6, 31]. Essential oils, characterized by multicomponent composition and multiple molecular targets, may reduce selective pressure for resistance development. In addition, plant-derived compounds generally exhibit lower environmental persistence due to volatility and photodegradation [32, 33], which may reduce long-term environmental accumulation. At the same time, potential environmental interactions must be considered. Essential oils and phenolic compounds may affect non-target arthropods present in pasture ecosystems. Therefore, further studies are necessary to evaluate possible short-term impacts on beneficial insects and dung-associated fauna under field conditions. Scalability of the proposed approach across different climatic conditions also requires consideration, as the activity of essential oils may vary with temperature, humidity, and parasite species composition [34]. In the present study, suppression of infestation indices persisted for approximately 14 days before the gradual reappearance of ectoparasites under continued grazing exposure. These results indicate the practical applicability of *N. cataria*–based formulations for short-term ectoparasite management in pasture systems. In addition, the possibility of local cultivation of *N. cataria* under Kazakh climatic conditions may further enhance the sustainability of this approach.

### Novelty and technological–biological integration

To our knowledge, this study represents the first field-based integration of a *N. cataria* essential oil-based biopreparation formulated with rice husk pyrolysis-derived plant liquids and delivered through an automated low-stress spraying system incorporating RFID identification and cumulative behavioral dosing in grazing cattle. While previous studies have largely focused on laboratory bioassays or short-term manual application of *N. cataria* extracts, no published reports have described integration of plant-based insecticidal formulations with automated precision delivery systems under pasture conditions. This technological–biological integration may represent a promising strategy for sustainable ectoparasite management in organic and low-input livestock production systems.

### Limitations and future research

The absence of an untreated field control group limits direct causal attribution of the observed efficacy exclusively to the tested formulation. Nevertheless, the complete suppression of detectable infestation (AI = 0) across all recorded parasite species during peak seasonal activity suggests that the observed effect was unlikely to be explained solely by natural seasonal fluctuations. Future randomized controlled trials including untreated and formulation-only control groups will be necessary to confirm these findings. Additional limitations should also be considered. Direct assessment of blood-feeding inhibition was not performed, which limits differentiation between insecticidal and repellent effects. The post-treatment observation period was relatively short and therefore does not allow conclusions regarding long-term persistence of the formulation. Furthermore, the trial was conducted in a single geographic region and during a single grazing season, which may limit the generalizability of the results. Environmental variables such as temperature, humidity, rainfall, and natural fluctuations in ectoparasite populations may also have influenced the observed outcomes despite efforts to standardize observation conditions.

## CONCLUSION

The present study demonstrated that an automated cumulative dosing system delivering an *N. cataria*–based biopreparation effectively controlled ectoparasites in cattle under pasture conditions. Laboratory evaluation confirmed a concentration-dependent insecticidal effect against major ectoparasite groups, while field application resulted in complete suppression of detectable infestation (AI = 0; OI = 0%) during the first 7–14 days following completion of the programmed cumulative dose. Partial re-infestation was observed by day 21, with efficacy ranging from 66.7% to 88.6% depending on parasite species. Importantly, physiological, hematological, and biochemical parameters remained within normal reference ranges, indicating that the treatment was safe under the tested conditions.

From a practical perspective, integrating plant-based insecticidal formulations with automated spraying technology offers a stress-free, labor-efficient, and precise approach to ectoparasite control in grazing livestock. The system enables individualized and cumulative dosing through voluntary animal movement, reducing handling stress and ensuring uniform treatment coverage. This approach is particularly relevant for organic and low-input livestock production systems, where the use of synthetic chemicals is restricted and environmentally sustainable alternatives are required.

A key strength of this study lies in the successful field-level integration of a plant-derived biopreparation with a precision livestock delivery system, representing a novel technological–biological strategy. The use of cumulative dosing under real pasture conditions, combined with comprehensive evaluation of efficacy and safety, enhances the translational value of the findings. Additionally, the formulation’s dual composition, incorporating both *N. cataria* essential oil and pyrolysis-derived plant compounds, suggests potential synergistic effects that may contribute to its biological activity.

However, the absence of an untreated control group, the relatively short observation period, and the lack of direct differentiation between insecticidal and repellent effects should be considered when interpreting the results. Future studies should include controlled experimental designs, extended monitoring periods, and mechanistic investigations to better understand formulation dynamics, long-term efficacy, and environmental interactions.

In conclusion, the automated application of an *N. cataria*–based biopreparation represents a promising, environmentally compatible, and technologically feasible strategy for short-term ectoparasite management in pasture-based cattle systems. The approach has significant potential to improve animal welfare, reduce reliance on synthetic insecticides, and advance sustainable livestock production practices.

## DATA AVAILABILITY

The datasets generated during the current study are available upon reasonable request from the corresponding author.

## AUTHORS’ CONTRIBUTIONS

GY: Data curation, formal analysis, methodology, resources, supervision, and writing – original draft. RU: Formal analysis, methodology, resources, project administration, supervision, and funding acquisition. SB: Project administration, resources, supervision, and funding acquisition. IT: Data curation, software, and visualization. AMu: Methodology and conceptualization. AMi: Methodology and conceptualization. FZ: Formal analysis, methodology, resources, visualization, and writing – review and editing. LL: Data curation, conceptualization, methodology, formal analysis, project administration, and writing – original draft. All authors have read and approved the final version of the manuscript.
